# Case Report: Acute angle-closure glaucoma as the initial presentation of autosomal recessive bestrophinopathy caused by compound heterozygous *BEST1* mutations

**DOI:** 10.3389/fmed.2026.1861665

**Published:** 2026-09-08

**Authors:** Ruiqi Lu, Lujia Zhou, Li Tang, Xiumiao Li, Guofan Cao

**Affiliations:** 1 The Affiliated Eye Hospital of Nanjing Medical University, Nanjing, China; 2 The Fourth School of Clinical Medicine, Nanjing Medical University, Nanjing, China

**Keywords:** angle-closure glaucoma (ACG), autosomal recessive bestrophinopathy(ARB), *BEST1* gene, case report, ultrasound cycloplasty (UCP)

## Abstract

**Background:**

Autosomal recessive bestrophinopathy (ARB) arises from compound heterozygous *BEST1* mutations. Beyond retinal pathology, it may be associated with short axial length, shallow anterior chamber, and refractory angle-closure glaucoma (ACG). Filtration surgery carries a high recurrence rate and may yield suboptimal outcomes in selected *BEST1*-associated eyes, with the optimal surgical strategy remaining undefined.

**Case presentation:**

A 29-year-old female presented with refractory angle-closure glaucoma. Clinical examination revealed bilateral elevated intraocular pressure (IOP) and shallow anterior chambers. Retinal imaging demonstrated macular clefting and subretinal fluid. Ocular history was notable for repeated trabeculectomies, which provided transient IOP control. Genetic testing identified compound heterozygous *BEST1* variants (c.922A > G and c.1740-1G > A). Familial segregation analysis demonstrated that the two *BEST1* variants were inherited from different parents and were present in trans, supporting the molecular diagnosis of ARB. Given the patient's short axial length and history of failed filtration surgeries, ultrasound cycloplasty (UCP) was performed on the left eye. Postoperatively, IOP in the left eye remained stable. At the 7-month follow-up, OCT showed a concurrent reduction in macular schisis-like changes and subretinal fluid in the UCP-treated eye, whereas these retinal abnormalities progressed in the medically managed fellow eye.

**Conclusion:**

This case represents a rare report of ARB presenting with refractory ACG in a patient harboring compound heterozygous *BEST1* variants, including c.922A > G, classified as likely pathogenic, and c.1740-1G > A, classified as a variant of uncertain significance. Young patients with ACG accompanied by short axial length, a shallow anterior chamber, and unexplained retinal abnormalities may benefit from early evaluation for ARB, including genetic testing. UCP may offer a potential alternative to conventional filtration surgery for IOP control in selected patients with ARB and refractory ACG. However, the theory requires confirmation in larger studies with longer follow-up.

## 1 Introduction

The *BEST1* gene encodes Bestrophin-1, which is a multifunctional protein localized to the basolateral membrane of the retinal pigment epithelium (RPE). Bestrophin-1 functions primarily as a calcium-activated chloride channel and a regulator of voltage-dependent calcium channels (1–3). It is essential for RPE ion homeostasis and fluid transport. ARB is caused by biallelic pathogenic variants in the *BEST1* gene. First described by Burgess et al. in 2008, the clinical hallmarks include vitelliform or multifocal macular deposits, subretinal and cystoid exudates, vitreoretinal and choroidal structural abnormalities and progressive visual impairment (4–7).

Recent clinical consensus indicates that the pathology of bestrophinopathies extends beyond the posterior segment. Many patients also exhibit anterior segment abnormalities, including high hyperopia, short axial length, and shallow anterior chamber (8). This anatomical crowding elevates the risk of ACG (6, 9), which is now recognized as a severe complication across the entire *BEST1*-related disease spectrum (5, 10). Previous reports indicate that traditional filtering procedures, such as trabeculectomy, carry a high risk of severe postoperative complications in this population. These complications include shallower anterior chamber, choroidal effusion, and malignant glaucoma(MG) (10–13). Conventional filtration surgery may not be a safe first-line option for these patients.

We report a Chinese female with compound heterozygous *BEST1* mutations who presented with early-onset refractory ACG, congenital-like retinal clefting, and macular edema. Following multiple failed trabeculectomies, she underwent UCP. Her IOP stabilized postoperatively, and OCT obtained at the 7-month follow-up showed concurrent improvement in macular schisis-like changes and subretinal fluid. This case provides insights into the diagnosis and surgical management of *BEST1*-associated ACG.

## 2 Case presentation

### 2.1 Patient background and initial presentation

**Figure 1:**
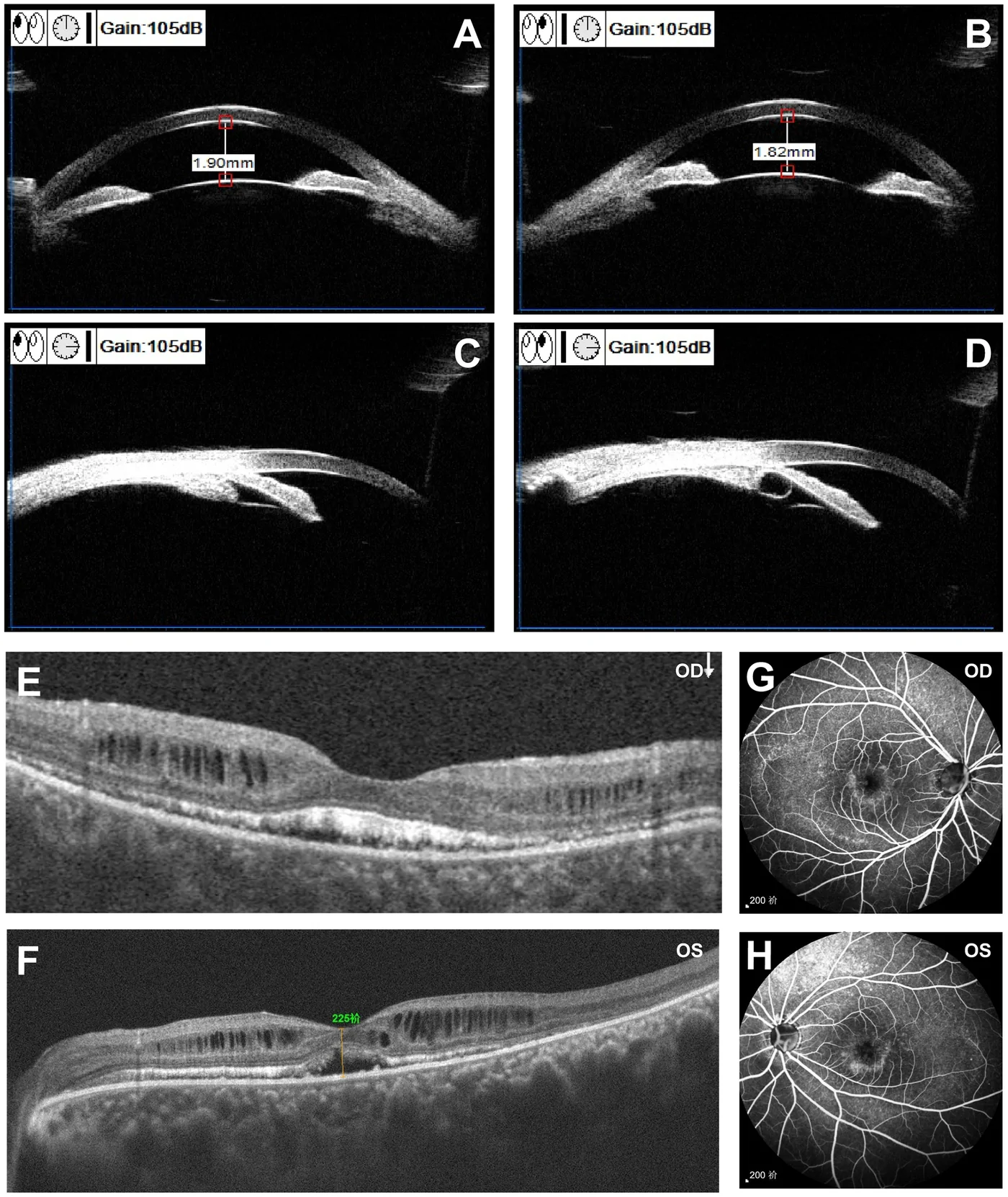
Ocular imaging at initial presentation. **(A–D)** UBM shows crowded anterior segment with shallow anterior chambers and extensive angle closure bilaterally. In the left eye, anterior rotation of the ciliary processes and a ciliary body cyst contribute to the anterior displacement of the lens-iris diaphragm. **(E,F)** OCT reveals characteristic macular schisis and localized subretinal fluid accumulation in both maculae. **(G,H)** Fundus fluorescein angiography (FFA) displays optic disc hypofluorescence indicating capillary nonperfusion. The macular region exhibits a ring-like pattern of transmission hyperfluorescence corresponding to RPE atrophy, with late-phase staining.

A 29-year-old female presented in 2020 with a 3-month history of bilateral visual decline, intermittent ocular discomfort, and sensation of ocular fullness. She reported subjective visual field narrowing but denied ocular redness, headache, nausea, vomiting, or systemic symptoms. At admission, best-corrected visual acuity (BCVA) was 0.6 in the right eye and 0.04 in the left eye. IOP was elevated bilaterally (30 mmHg right; 33 mmHg left). Slit-lamp examination and gonioscopy revealed bilateral shallow anterior chambers and predominantly closed angles. Ultrasound biomicroscopy (UBM) further demonstrated bilateral shallow anterior chambers and abnormal angle structures. Optical coherence tomography (OCT) showed generalized peripapillary retinal nerve fiber layer (RNFL) thinning, macular schisis-like changes and localized subretinal fluid. Visual field testing confirmed extensive central and peripheral defects bilaterally (Figure 1). Based on these findings, preliminary diagnoses included bilateral AACG, optic nerve atrophy, congenital-like retinal clefts, and macular RPE lesions.

### 2.2 Review of clinical course and follow-up evaluation

The patient's clinical course was characterized by refractory bilateral chronic angle-closure glaucoma. In 2020, recurrent IOP elevation prompted sequential trabeculectomy in both eyes. Routine postoperative anti-inflammatory treatment was administered after each procedure. However, postoperative IOP control was transient, and filtration function progressively failed. The patient continued topical IOP-lowering treatment in both eyes. The regimen consisted of brimonidine tartrate eye drops, tafluprost eye drops, and brinzolamide–timolol (a fixed combination of brinzolamide and timolol). Approximately 5 months after the two trabeculectomy procedures, the patient reported progressive visual decline in both eyes, with occasional ocular fullness and discomfort. Bilateral complicated cataracts were diagnosed. Phacoemulsification with intraocular lens implantation was subsequently performed in both eyes. In 2023, IOP in the right eye surged to approximately 40 mmHg. A second trabeculectomy was therefore performed in the right eye. Routine postoperative anti-inflammatory treatment was administered, and the same topical IOP-lowering regimen was continued. At the re-evaluation in December 2024, IOP was 19 mmHg in the right eye and 29 mmHg in the left eye. The left-eye IOP was considered uncontrolled despite medical treatment. At that time, IOP in the right eye remained adequately controlled with topical medications after the second trabeculectomy.

**Figure 2:**
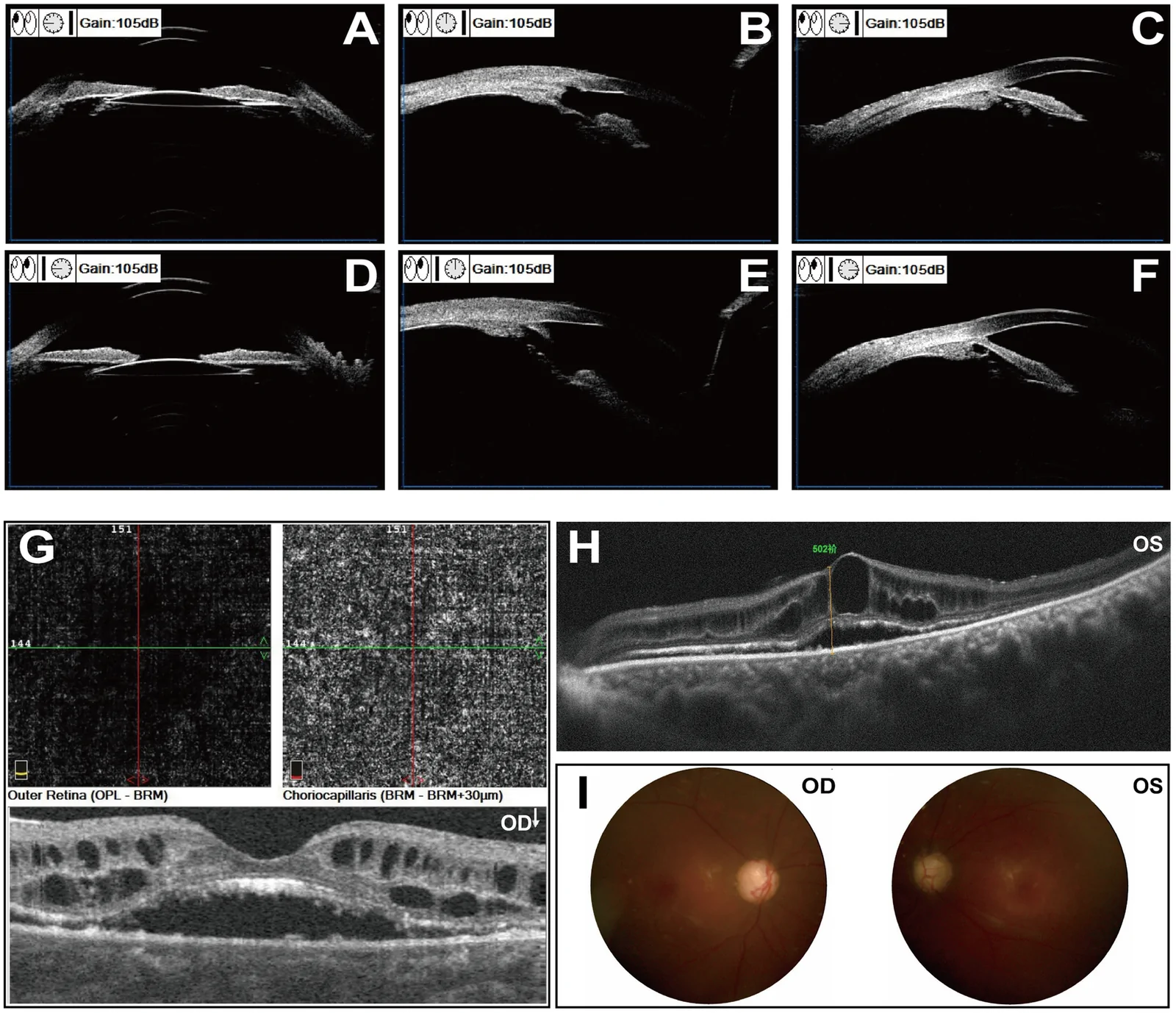
Ophthalmic imaging after three trabeculectomy procedures in total: two in the right eye and one in the left eye. **(A–F)** UBM shows bilateral intraocular lenses, complete angle closure, anterior iris prolapse morphology, and a persistent ciliary body cyst in the left eye. **(G,H)** OCT and OCT angiography (OCTA) demonstrate extensive retinal schisis-like changes, cystic spaces, and subfoveal subretinal detachment bilaterally (central macular thickness: OD, 374 μm; OS, 502 μm). **(I)** Fundus photography reveals increased CDR and characteristic yellowish-white subretinal deposits bilaterally.

Comprehensive re-evaluation in December 2024 revealed markedly short axial lengths (OD: 20.96 mm; OS: 20.79 mm) and shallow anterior chambers bilaterally. It indicates severe anterior segment crowding. Fundus photography demonstrated advanced glaucomatous optic neuropathy, characterized by severe peripapillary RNFL thinning and cup-to-disc ratio (CDR) ratios approaching 1.0 bilaterally. Macular evaluation revealed retinal schisis-like changes and serous subretinal fluid in both eyes (Figure 2).

The combination of anterior segment crowding, highly refractory glaucoma, and atypical macular pathology suggested an underlying hereditary etiology. Genetic testing was consequently ordered to clarify the diagnosis and guide future treatment strategies.

### 2.3 Molecular diagnosis and familial segregation analysis

**Figure 3:**
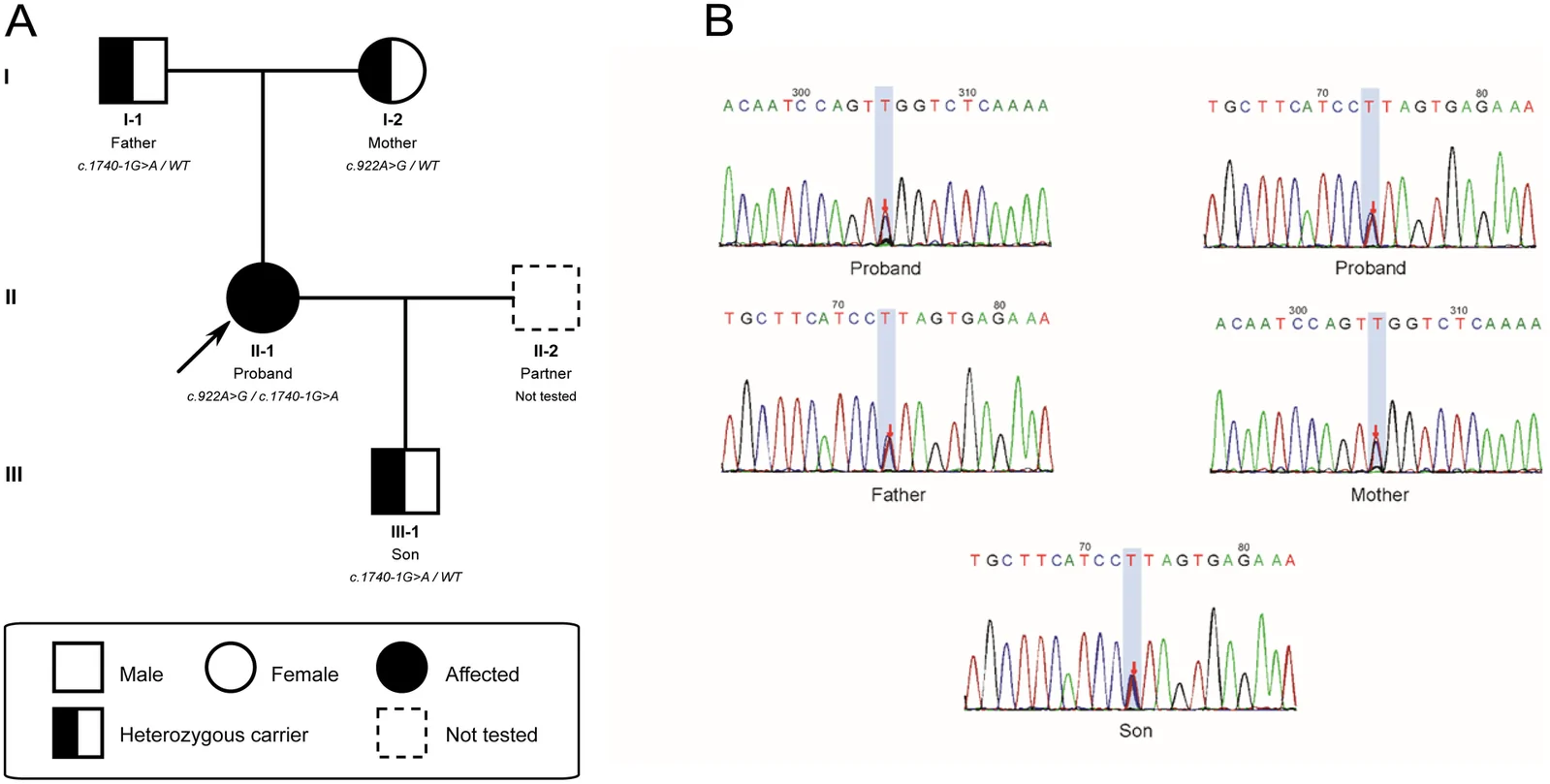
Pedigree of the family and Sanger validation of the two *BEST1* variants. **(A)** The proband (arrow) carries compound heterozygous mutations in the *BEST1* gene. Filled symbols indicate affected individuals; half-filled symbols indicate heterozygous carriers. **(B)** The proband carries compound heterozygous mutations, c.922A > G (p.Asn308Asp) (derived from the mother) and c.1740-1G > A (derived from the father). The proband's son carries only the heterozygous c.1740-1G > A variant, and both the proband's parents and son exhibit normal phenotypes. The segregation pattern supports a trans configuration and is consistent with autosomal recessive inheritance.

Next generation sequencing and sanger sequencing of family members showed that the proband carried compound heterozygous mutations in the *BEST1* gene, c.922A > G (p.Asn308Asp) (derived from the mother) and c.1740-1G > A (derived from the father) (Table 1, Figure 3). Neither variant has been reported in the literature within the HGMD database. The first variant, c.922A > G, results in the substitution of asparagine by aspartic acid at amino acid position 308. According to the standards and guidelines for sequence variant interpretation established by the American College of Medical Genetics and Genomics, this variant was classified as a Likely Pathogenic mutation (PM1 + PM2_Supporting+PM5 + PP3_Moderate). The second variant, c.1740-1G > A, is a canonical splice-site mutation. This mutation affects the acceptor splice site within intron 10 of the *BEST1* gene. Although this variant is not predicted to cause nonsense-mediated decay (NMD), it is highly likely to alter RNA splicing and lead to disruption of the protein product. According to ACMG guidelines, this mutation was preliminarily assessed as a Variant of Uncertain Significance (PVS1_Moderate+PM2_Supporting + PM3). Additionally, the proband's son carries only the heterozygous c.1740-1G > A variant, and both the proband's parents and son exhibit normal phenotypes. This indicates co-segregation of genotype and clinical phenotype, consistent with autosomal recessive inheritance. Given the combination of her clinical phenotype and biallelic *BEST1* mutations, we confirmed a diagnosis of ARB. This prompted a critical reassessment of her surgical risks and overall glaucoma treatment strategy.

**Table 1:** Molecular characteristics and familial segregation of the BEST1 variants identified in the proband and family members.

Member	gene	Chromosome position	Transcript exon	Nucleotide change	Amino acid change	Zygosity	Pathogenicity	Associated disease reference	Source of variation
patient	*BEST1*	chr11:617 27024	NM_0041 83.4; exon 8	c.922A > G	p.Asn308Asp	het	Likely pathogenic	ARB	mother
	*BEST1*	chr11:617 31575	NM_0041 83.4; intro n10	c.1740-1G > A	p.?	het	Uncertain	ARB	father
father	*BEST1*	chr11:617 31575	NM_0041 83.4; intro n10	c.1740-1G > A	p.?	het	Uncertain	ARB	
mother	*BEST1*	chr11:617 27024	NM_0041 83.4; exon 8	c.922A > G	p.Asn308Asp	het	Likely pathogenic	ARB	
son	*BEST1*	chr11:617 31575	NM_0041 83.4; intro n10	c.1740-1G > A	p.?	het	Uncertain	ARB	

*BEST1* mutations of genetic testing results ARB, autosomal recessive bestrophinopathy.

### 2.4 Treatment strategy adjustment and outcomes

Following molecular diagnosis of ARB, we re-evaluated the patient's management. Given her short axial length, shallow anterior chambers and multiple failed trabeculectomies, we believe that the success rate of further filtration procedures is low. Consequently, we pivoted to a strategy of reducing aqueous humor production. The left eye underwent UCP. At discharge after UCP, brimonidine tartrate eye drops were discontinued. Levofloxacin eye drops were prescribed three times daily in the left eye. Pranoprofen eye drops were also prescribed three times daily in the left eye. Brinzolamide–timolol was prescribed twice daily in both eyes. Tafluprost eye drops were prescribed once nightly in both eyes. Levofloxacin and pranoprofen were given as routine postoperative treatment. Brinzolamide–timolol and tafluprost were prescribed for IOP control. None of these medications was prescribed specifically for macular schisis or subretinal fluid. Levofloxacin and pranoprofen were continued for 2 weeks. Brinzolamide–timolol and tafluprost were continued throughout the follow-up period.

**Figure 4:**
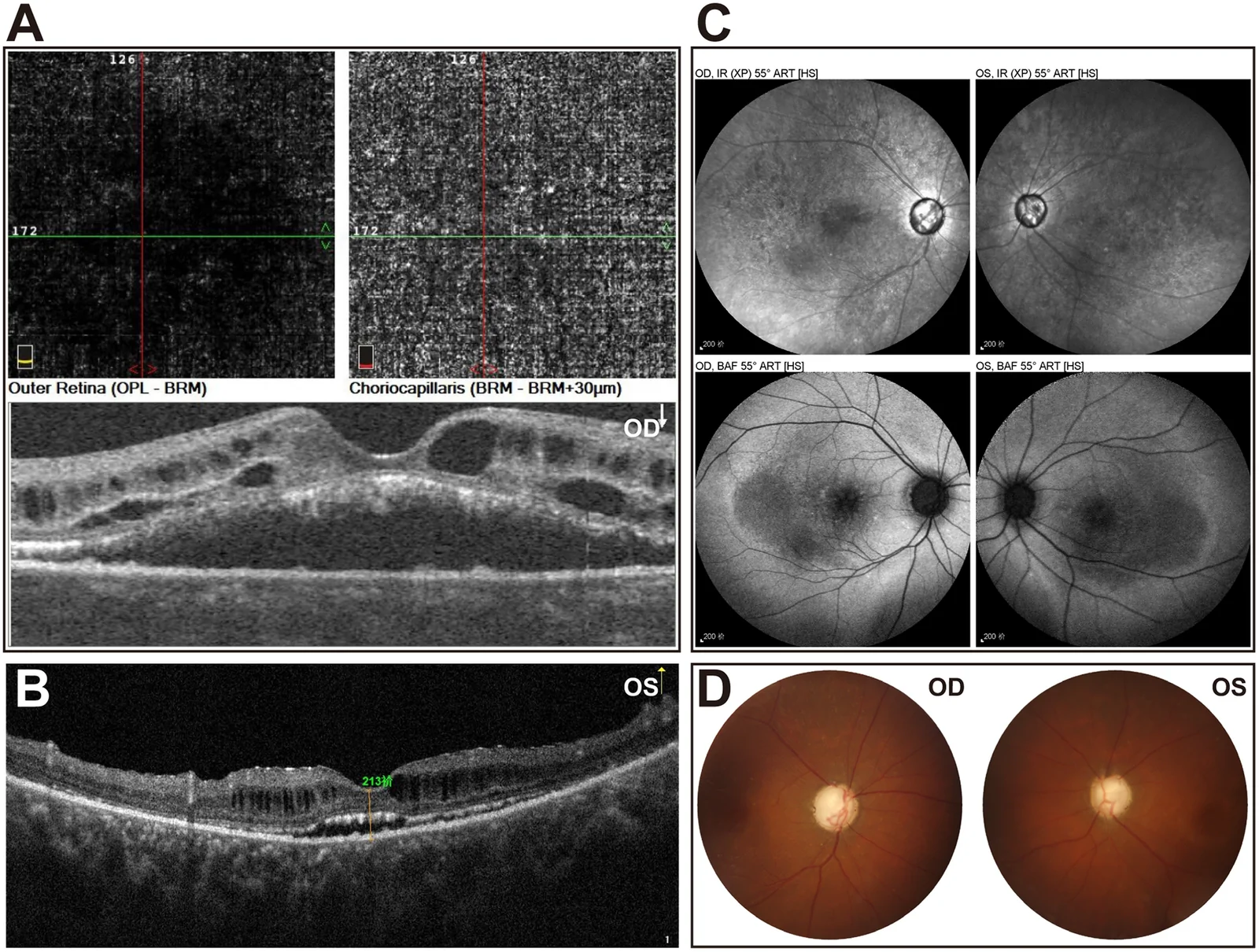
Postoperative ocular imaging at 7 months. **(A,B)** OCT and OCTA show the resolution of macular edema and schisis-like changes in the UCP-treated left eye (CMT reduced to 213 μm). The medically managed right eye exhibits disease progression (CMT increased to 451 μm). **(C)** Fundus autofluorescence shows mixed hyper- and hypoautofluorescence, consistent with ARB, distinct from classic Best vitelliform macular dystrophy (BVMD) lesions (13). **(D)** Fundus photography documents stable advanced glaucomatous optic neuropathy with CDR approaching 1.0.

Postoperatively, the left eye remained stable without severe complications such as MG or hypotony. At 7 months after UCP, OCT showed a marked reduction in macular schisis-like changes and subretinal fluid in the left eye. CMT decreased from 502 μm to 213 μm. In contrast, the fellow right eye, which did not undergo UCP, showed progression of macular schisis-like changes and fluid accumulation. CMT increased from 374 μm to 451 μm (Figure 4). Anterior segment slit-lamp photographs obtained 2 days before and 1 week after UCP are provided in Supplementary Figures S1, S2, respectively.

These comparative outcomes suggest that UCP is a safe and effective intervention for refractory IOP in *BEST1*-associated glaucoma. Beyond intraocular pressure control, UCP may provide additional therapeutic benefits. It reduces aqueous humor production and modulates intraocular hemodynamics. These effects may alleviate mechanical or osmotic stress on the RPE–retina interface. Subretinal fluid absorption and macular schisis resolution may be promoted in patients with ARB.

## 3 Discussion

This case represents a rare report of an association between ARB complicated by refractory ACG and compound heterozygous mutations in *BEST1* (c.922A > G and c.1740-1G > A). The key to this case lies in the genetic confirmation of ARB through these *BEST1* compound heterozygous mutations, with clinical findings consistent with a typical ARB phenotype. The splice-site variant c.1740-1G > A remains a VUS under ACMG guidelines. However, in the context of a compatible ARB phenotype and segregation analysis, it was considered a likely splice-altering, disease-contributing variant. Familial Sanger sequencing demonstrated that the two variants were inherited from different parents, supporting a trans configuration. In this family, the proband's mother carried only the heterozygous c.922A > G variant and had no clinically apparent ocular phenotype. The proband's father and son carried only the heterozygous c.1740-1G > A variant and were also phenotypically normal. Whole-exome/whole-genome sequencing and functional studies, including patient-derived RNA analysis, minigene splicing assays, and protein functional analyses, were not performed in this case. The functional effects of both *BEST1* variants could not be directly confirmed in this patient. In addition, deep intronic, regulatory, and other genomic variants outside the targeted sequencing regions were not comprehensively evaluated. Previous cohort data showed that patients with homozygous or compound heterozygous *BEST1* variants had shorter axial lengths, shallower anterior chambers, and higher IOP than patients with heterozygous variants or without *BEST1* variants (14). Biallelic *BEST1* variants may be associated with a broader ocular phenotype involving both the posterior and anterior segments (14).

The therapeutic recalcitrance of this case is rooted in congenital anatomical anomalies. Short axial length, a hallmark of bestrophinopathies, results in severe anterior segment crowding (7). This immutable anatomical substrate largely dictated the poor surgical outcomes. Performing trabeculectomy on such high-risk anatomy may increase the risk of complications including shallow anterior chamber, excessive inflammation, and filtration failure. Vitreous abnormalities further amplify these risks. The vitreous plays a pivotal role in both the pathogenesis of ACG and the high incidence of postoperative MG in this population (11). *BEST1* mutations are associated with persistent “solid and unliquefied vitreous”, which induces high posterior chamber pressure. This anterior vector force displaces the lens-iris diaphragm forward, precipitating secondary angle closure (11).

*BEST1* dysfunction compromises the RPE fluid pump, preventing effective fluid transport from the subretinal space to the choroid and leading to chronic macular edema. In these patients, the sudden decompression associated with trabeculectomy disrupts IOP equilibrium. Given solid and unliquefied vitreous and elevated posterior chamber pressure typical of ARB, this decompression creates a steep anterior-posterior gradient, triggering acute choroidal expansion or effusion. The resulting anterior displacement of the lens-iris diaphragm causes ciliary block and anterior chamber flattening, ultimately obstructing the filtration ostium. Vitreous abnormalities likely underpin both posterior retinoschisis and anterior angle closure (15). This suggests that recurrent ocular hypertension and vitreoretinal and choroidal complex abnormalities share a common genetic etiology.

The cumulative failure of three trabeculectomies in our patient aligns with the high-risk anatomical profile identified by Fang et al., (mean axial length 21.53 mm) (11). They reported a 100% incidence of MG following standalone trabeculectomy in *BEST1* patients due to nanophthalmos and shallow anterior chambers. Conversely, combined procedures (phacoemulsification-IOL-trabeculectomy plus anterior pars plana vitrectomy) were shown to prevent MG (11). Similarly, another cohort reported universal MG onset post-filtration surgery, suggesting that standard filtration is contraindicated in this population, irido-zonulo-hyaloid-vitrectomy or pars plana vitrectomy may be considered in selected high-risk eyes (10).

Patients with ARB often have a short axial length, a crowded anterior segment, and abnormal vitreous structure. These features increase the risk of postoperative anterior chamber shallowing and MG. Although our patient did not exhibit acute MG, the limited durability of IOP control after three trabeculectomy procedures suggests that the patient's short axial length and crowded anterior segment may have contributed to the unfavorable filtration outcomes. However, the possible effects of surgical technique, wound healing, and postoperative bleb management cannot be excluded. Consequently, upon confirming the ARB diagnosis, we selected UCP after an individualized assessment.

A previous case report suggested that low-dose transscleral cyclophotocoagulation (TCP) may alleviate both ACG and retinoschisis by inducing vitreous liquefaction (15). Unlike traditional TCP, which utilizes laser-induced photothermal coagulation and can cause significant collateral tissue damage or uveitis, UCP employs high-intensity focused ultrasound (HIFU) (16–18). This technology allows for precise, computer-controlled ablation of the ciliary body epithelium with minimal impact on surrounding sclera or conjunctiva (16–18). By reducing aqueous humor production without creating an additional filtering fistula, UCP may provide an alternative IOP-lowering option for selected eyes with a crowded anterior segment (19–21).

Beyond IOP control, UCP may have indirectly contributed to the retinal improvement in this patient. Studies suggest that UCP may modify aqueous dynamics by reducing aqueous production and increasing uveoscleral outflow (22–24). UCP may also change anterior segment configuration in eyes with angle closure (23). We hypothesize that these effects created a more stable pressure-related biomechanical environment in the treated eye. This effect may be relevant in ARB eyes with a short axial length, crowded anterior segment, and abnormal vitreous structure. A more stable ocular environment may reduce mechanical stress at the vitreoretinal interface. It may therefore favor a reduction in macular schisis-like changes and subretinal fluid. However, the temporal association observed in this case does not establish causality. The possible effects of postoperative pranoprofen and brinzolamide cannot be excluded. Natural fluctuations in ARB-associated retinal fluid are also possible. Because the two eyes differed in baseline visual and retinal severity, previous surgical treatment, and IOP control at the time of UCP, the fellow eye should not be regarded as a strictly comparable internal control. Further studies are required to evaluate the relationship between UCP, ocular biomechanics, and retinal anatomy.

ARB must be considered in the differential diagnosis for any patient under 40 years presenting with bilateral angle-closure glaucoma, short axial length, and unexplained maculopathy or retinoschisis. Preoperative *BEST1* screening is clinically imperative in such cases. As proposed by Low et al., surgical strategy (e.g., primary PPV with tube shunt) is dictated by genetic diagnosis. The surgico-genetics paradigm can effectively prevent MG and ensure long-term IOP control (25). Definitive genetic diagnosis is essential not only for predicting surgical risk but also for defining optimal long-term management.

## 4 Conclusions

By integrating longitudinal clinical data with rigorous genetic analysis, we confirm *BEST1* compound heterozygous mutations as the etiology of refractory angle-closure glaucoma and complex retinal pathology in this patient, establishing a diagnosis of ARB. This case highlights the potential risk in selected patients and underscores the critical necessity of genetic screening for young ACG patients. Implementing such protocols is essential for achieving precision diagnosis and ensuring safer, personalized therapeutic strategies. Although the precise pathogenesis of ARB and the long-term efficacy of UCP require further investigation, UCP may be considered as an alternative IOP-lowering option in selected patients with inadequate medical control and a high risk associated with repeat filtration surgery. Further studies with larger samples and longer follow-up are required.

## Data Availability

The raw targeted gene panel sequencing data generated in this study have been deposited in the Genome Sequence Archive for Human (GSA-Human) at the National Genomics Data Center, China National Center for Bioinformation, under accession number HRA020576. Due to ethical and privacy restrictions associated with human genomic data, access to the sequencing data is controlled. Qualified researchers may apply for access through the GSA-Human data access system, subject to approval by the corresponding Data Access Committee. The dataset is available at https://ngdc.cncb.ac.cn/gsa-human/browse/HRA020576.

## References

[1] Sun H Tsunenari T Yau K-W Nathans J. The vitelliform macular dystrophy protein defines a new family of chloride channels. Proc Natl Acad Sci USA. (2002) 99:4008–13. 10.1073/pnas.052692999 11904445 PMC122639

[2] Qu Z Cheng W Cui Y Cui Y Zheng J. Human disease-causing mutations disrupt an N-C-terminal interaction and channel function of bestrophin 1. J Biol Chem. (2009) 284:16473–81. 10.1074/jbc.M109.002246 19372599 PMC2713530

[3] Rosenthal R Bakall B Kinnick T Peachey N Wimmers S Wadelius C. Expression of bestrophin-1, the product of the *VMD2* gene, modulates voltage-dependent Ca2+ channels in retinal pigment epithelial cells. FASEB J. (2006) 20:178–80. 10.1096/fj.05-4495fje 16282372

[4] Burgess R Millar ID Leroy BP Urquhart JE Fearon IM De Baere E. Biallelic mutation of *BEST1* causes a distinct retinopathy in humans. Am J Hum Genet. (2008) 82:19–31. 10.1016/j.ajhg.2007.08.004 18179881 PMC2253971

[5] Kim HR Han J Kim YJ Kang HG Byeon SH Kim SS. Clinical features and genetic findings of autosomal recessive bestrophinopathy. Genes (Basel). (2022) 13:1197. 10.3390/genes13071197 35885980 PMC9320462

[6] Casalino G Khan KN Armengol M Wright G Pontikos N Georgiou M. Autosomal recessive bestrophinopathy: clinical features, natural history, and genetic findings in preparation for clinical trials. Ophthalmology. (2021) 128:706–18. 10.1016/j.ophtha.2020.10.006 33039401 PMC8062850

[7] Pfister TA Zein WM Cukras CA Sen HN Maldonado RS Huryn LA. Phenotypic and genetic spectrum of autosomal recessive bestrophinopathy and best vitelliform macular dystrophy. Invest Ophthalmol Visual Sci. (2021) 62:22. 10.1167/iovs.62.6.22 PMC8142704 34015078

[8] Boon CJF Van Den Born LI Visser L Keunen JEE Bergen AAB Booij JC. Autosomal recessive bestrophinopathy: differential diagnosis and treatment options. Ophthalmology. (2013) 120:809–20. 10.1016/j.ophtha.2012.09.057 23290749

[9] Low S Davidson AE Holder GE Hogg CR Bhattacharya SS Black GC. Autosomal dominant best disease with an unusual electrooculographic light rise and risk of angle-closure glaucoma: a clinical and molecular genetic study. Mol Vision. (2011) 17:2272–82. 21921978 PMC3171497

[10] Parameswarappa DC Balasubramnian J Kumar Padhy S Hansraj S Natarajan R Kannabiran C. *BEST1* Associated bestrophinopathies with angle closure and post-surgical malignant glaucoma. Ophthalmic Genet. (2024) 45:571–82. 10.1080/13816810.2024.2398827 39259030

[11] Fang Y Duan X Chen L Shi J Liu J Sun Y. Combination of trabeculectomy and primary pars plana vitrectomy in the successful treatment of angle-closure glaucoma with *BEST1* mutations: self-controlled case series. Ophthalmol Ther. (2022) 11:2271–84. 10.1007/s40123-022-00580-1 36223057 PMC9587171

[12] Zhong Y Guo X Xiao H Luo J Zuo C Huang X. Flat anterior chamber after trabeculectomy in secondary angle-closure glaucoma with *BEST1* gene mutation: case series. PLoS One. (2017) 12:e0169395. 10.1371/journal.pone.0169395 28056057 PMC5215797

[13] Sun Z Liu C Liu W Zhang M Ren S Gao L. Surgical treatment of autosomal recessive bestrophinopathy with angle-closure glaucoma: vitreous liquefaction as the key to correcting postoperative malignant glaucoma—three case reports. Front Med. (2025) 12:1560475. 10.3389/fmed.2025.1560475 PMC12615444 41244779

[14] Xuan Y Zhang Y Zong Y Wang M Li L Ye X. The clinical features and genetic spectrum of a large cohort of Chinese patients with vitelliform macular dystrophies. Am J Ophthalmol. (2020) 216:69–79. 10.1016/j.ajo.2020.03.047 32278767

[15] Shi Y Tian J Han Y Oatts J Wang N. Pathogenic role of the vitreous in angle-closure glaucoma with autosomal recessive bestrophinopathy: a case report. BMC Ophthalmol. (2020) 20:271. 10.1186/s12886-020-01543-5 32646389 PMC7346445

[16] Yang L Gao J Yan Y Shi G. Impact of ultrasound cycloplasty on ocular biomechanics and refractive parameters: a comprehensive review. Front Med. (2026) 13:1783195. 10.3389/fmed.2026.1783195 PMC12953110 41783060

[17] Wang T Wang R Su Y Li N. Ultrasound cyclo plasty for the management of refractory glaucoma in Chinese patients: a before–after study. Int Ophthalmol. (2021) 41:549–58. 10.1007/s10792-020-01606-y 33070270

[18] Yu Q Liang Y Ji F Yuan Z. Comparison of ultrasound cycloplasty and transscleral cyclophotocoagulation for refractory glaucoma in Chinese population. BMC Ophthalmol. (2020) 20:387. 10.1186/s12886-020-01655-y 32993561 PMC7525941

[19] Marques RE Ferreira NP Sousa DC Barata AD Sens P Marques-Neves C. High intensity focused ultrasound for glaucoma: 1-year results from a prospective pragmatic study. Eye (Lond). (2021) 35:484–9. 10.1038/s41433-020-0878-0 32317796 PMC8027663

[20] Chen MF Kim CH Coleman AL. Cyclodestructive procedures for refractory glaucoma. Cochrane Database Syst Rev. (2019) 3:CD012223. 10.1002/14651858.CD012223.pub2 30852841 PMC6409080

[21] Shi L Liu Y Wang R Shen W Yang J. Clinical efficacy of ultrasound cycloplasty for glaucoma patients and pathological observation on the ciliary body in rabbit models. J Ophthalmol. (2026) 2026:1279697. 10.1155/joph/1279697 42591775 PMC13463128

[22] Mastropasqua R Agnifili L Fasanella V Toto L Brescia L D Staso S. Uveo-scleral outflow pathways after ultrasonic cyclocoagulation in refractory glaucoma: an anterior segment optical coherence tomography and *in vivo* confocal study. Br J Ophthalmol. (2016) 100:1668–75. 10.1136/bjophthalmol-2015-308069 26883868

[23] Liu G Mao YK Liu NX Tan JK Qiao G Chen ZJ. Efficacy, safety and IOP-lowering mechanisms of ultrasound cycloplasty for angle-closure glaucoma. Int J Ophthalmol. (2025) 18(11):2079–88. 10.18240/ijo.2025.11.09 41158182 PMC12554540

[24] Aptel F Béglé A Razavi A Romano F Charrel T Chapelon J-Y. Short- and long-term effects on the ciliary body and the aqueous outflow pathways of high-intensity focused ultrasound cyclocoagulation. Ultrasound Med Biol. (2014) 40:2096–106. 10.1016/j.ultrasmedbio.2014.04.017 24996576

[25] Low S Mohamed R Davidson A Papadopoulos M Grassi P Webster AR. A new paradigm for delivering personalised care: integrating genetics with surgical interventions in *BEST1* mutations. Eye. (2020) 34:577–83. 10.1038/s41433-019-0553-5 31455904 PMC7042240

