# Supplementary material for: Case Report: Acute angle-closure glaucoma as the initial presentation of autosomal recessive bestrophinopathy caused by compound heterozygous BEST1 mutations
Source: Front Med (Lausanne). 2026 Sep 8;13:1861665. doi: 10.3389/fmed.2026.1861665 (PMC13593448; doi:10.3389/fmed.2026.1861665)
Supplement: Supplementary file 1 [file Datasheet1.docx]

Supplementary Material

## 1.1 Supplementary Figures

**Supplementary Figure 1.** Preoperative slit-lamp anterior segment photographs obtained 2 days before UCP. The images show bilateral pseudophakia, superior surgical peripheral iridectomies, and relatively shallow anterior chambers under narrow slit-beam illumination.

**Supplementary Figure 2.** Postoperative slit-lamp anterior segment photographs obtained 1 week after UCP. Compared with the preoperative images, postoperative slit-lamp photographs showed increased conjunctival and ciliary injection and a relatively dilated, mildly irregular pupil in the left eye, while the anterior chamber remained formed without obvious corneal edema. No substantial interval change was observed in the right eye.
